# Dynamic Space Allocation Based on Internal Demand for Optimizing Release of Shared Parking

**DOI:** 10.3390/s22010235

**Published:** 2021-12-29

**Authors:** Shuo-Yan Chou, Anindhita Dewabharata, Ferani Eva Zulvia

**Affiliations:** 1 Department of Industrial Management, National Taiwan University of Science and Technology, Taipei 106, Taiwan; sychou@mail.ntust.edu.tw (S.-Y.C.); d10101801@mail.ntust.edu.tw (A.D.); 2 Center for Cyber-Physical System Innovation, National Taiwan University of Science and Technology, Taipei 106, Taiwan; 3 Department of Logistics Engineering, Universitas Pertamina, Jakarta 12220, Indonesia

**Keywords:** shared parking, shared economy, prediction, recurrent neural network, intelligent transportation system, smart cities

## Abstract

The size of cities has been continuously increasing because of urbanization. The number of public and private transportation vehicles is rapidly increasing, thus resulting in traffic congestion, traffic accidents, and environmental pollution. Although major cities have undergone considerable development in terms of transportation infrastructure, problems caused by a high number of moving vehicles cannot be completely resolved through the expansion of streets and facilities. This paper proposes a solution for the parking problem in cities that entails a shared parking system. The primary concept of the proposed shared parking system is to release parking lots that are open to specific groups for public usage without overriding personal usage. Open-to-specific-groups parking lots consist of parking spaces provided for particular people, such as parking buildings at universities for teachers, staff, and students. The proposed shared parking system comprises four primary steps: collecting and preprocessing data by using an Internet of Things system, predicting internal demand by using a recurrent neural network algorithm, releasing several unoccupied parking lots based on prediction results, and continuously updating the real-time data to improve future internal usage prediction. Data collection and data forecasting are performed to ensure that the system does not override personal usage. This study applied several forecasting algorithms, including seasonal ARIMA, support vector regression, multilayer perceptron, convolutional neural network, long short-term memory recurrent neural network with a many-to-one structure, and long short-term memory recurrent neural network with a many-to-many structure. The proposed system was evaluated using artificial and real datasets. Results show that the recurrent neural network with the many-to-many structure generates the most accurate prediction. Furthermore, the proposed shared parking system was evaluated for some scenarios in which different numbers of parking spaces were released. Simulation results show that the proposed shared parking system can provide parking spaces for public usage without overriding personal usage. Moreover, this system can generate new income for parking management and/or parking lot owners.

## 1. Introduction

The rapidly increasing number of vehicles without a corresponding increase in the availability of parking lots is causing problems in several cities worldwide. Finding a parking lot is a common problem encountered daily by numerous people in cities [1,2]. This problem is severe and affects several aspects of human life. The lack of parking lots causes traffic congestion because people searching or waiting for an available parking lot move slowly, causing a long queue to form on streets and blocking other cars [3]. Moreover, cars moving slowly when searching for parking increases carbon emissions [4].

The problem of limited parking lots can be solved using two general approaches. The first approach includes building new parking lots. Although this solution would increase the number of parking lots, it causes other problems and is cost-intensive. The second solution is increasing the utilization rate of existing parking lots. Parking lots are available in places such as public buildings, office buildings, residential buildings, and streets. In general, open-to-specific-group parking lots are only available for particular people or communities. For example, parking lots at a school are only available for teachers, staff, and students at that school. Parking lots of an office building can only be used by people working in that building. Although such parking lots were originally built for the convenience of people working at a given facility, some of these lots have a considerable number of unused spaces. Therefore, rather than building new parking lots, this study proposes a system to solve this problem by increasing the utilization rate of open-to-specific-group parking lots as an embodiment of the sharing economy. The idea of sharing private parking spaces has been examined in some studies. Zhang et al. [5] studied the sharing of private residential parking spaces and analyzed potential characteristics of residents for sharing private parking spaces. Cui et al. [6] analyzed the efficiency of a shared parking system. Hu et al. [7] applied game theory to design shared parking policies. These studies indicate that a shared parking system is a favorable solution to the parking problem in cities.

The primary idea of the proposed shared parking system is to ensure that open-to-specific-group parking lots are available for public usage without overriding personal usage. Some applications, such as Pavemint (www.pavemint.com, accessed on 21 November 2019), mobypark (www.mobypark.com, accessed on 21 November 2019), and park-king (www.park-king.com, accessed on 21 November 2019), have experimented with shared private parking. The proposed shared parking system enhances the common shared parking system by embedding the demand prediction feature. Because open-to-specific-group parking lots are only available for certain people, a typical pattern of usage can be observed. The system analyzes this pattern, forecasts future personal or internal usage, and recommends releasing the available parking lots for public use. The system collects internal usage data from sensors installed on parking gates. Before releasing parking spaces to the public, the system predicts future internal usage for the next seven days by using a long short-term memory (LSTM) recurrent neural network (RNN) algorithm. Then, the system can make decisions regarding the number of parking spaces that can be released for public usage at each hour for the next seven days. To improve the accuracy of predictions, the prediction result is updated daily on the basis of the latest data. Therefore, if any abnormality occurs, the system can rapidly respond. A reservation system is used to control public utilization. The proposed features help the parking space owner to increase their profit by recommending a high potential time to release their parking space. On the other hand, the prediction feature can also help the driver better plan for their parking space need.

This study used parking lots owned by a university in Taipei City as a case study. The proposed shared parking system was verified using historical data from the university. The objective was to maximize profit and simultaneously maintain the level of service for internal users. The proposed system is suitable for cities with limited parking spaces and with some buildings with open-to-specific-group parking lots.

The remainder of this paper is organized as follows: Section 2 provides a brief review of some literature related to this paper. Section 3 presents the proposed system, and Section 4 discusses its applications and validation. Concluding remarks are provided in Section 5.

## 2. Literature Review

This section briefly reviews some basic theories applied in this study, including those concerning recent parking problems, shared parking systems, and forecasting using the RNN.

### 2.1. Shared Parking System

Parking is an essential requirement in many cities. The increasing number of vehicles, which has not been followed by an increase in the number of parking spaces, has caused numerous parking problems. A shared parking system has emerged as an alternative solution to the parking problem in cities [8]. Abbott and Bigazzi [9] showed the shared parking system could cut down on-street parking congestion and mitigate uneven supply. Additionally, shared parking increases the occupancy rate while reducing overall supply and land use [10]. It can reduce 10% to 30% of the need for parking development [11]. 

In general, private parking is used occasionally or during some specific hours. Therefore, for the remaining hours of the day, a parking space can be used by other people who require it. Thus, it needs a platform to provide a system that allows users to find and rent an available parking space. Shao et al. [12] developed the initial reservation and allocation system for shared parking. On the other hand, some parking applications—such as Pavemint (www.pavemint.com, accessed on 21 November 2019), mobypark (www.mobypark.com, accessed on 21 November 2019), and park-king (www.park-king.com, accessed on 21 November 2019)—have employed the aforementioned system. On these platforms, parking space owners must specify the available time for public usage. 

Although this system might be able to solve the parking problem in cities, some problems may occur with implementation. The implementation is closely related to the supply and demand mechanism—i.e., the building or parking owners as the suppliers and the public or car driver as the demanders [13]. Cui, Guo, Zhao, and Xi [6] analyzed some concerns related to supply, demand, and acceptable walking distance. Therefore, Cai et al. [14] built a method to have the proper amount of profit by balancing the benefit of parking lots and maintaining the requirement of building users. Zhao et al. [15] developed intelligent parking management systems to simulate the operation of shared parking considering the uncertainties of public users’ and the owner users’ arrival and departure using an agent-based model. Moreover, shared parking can provide potential flow income for public, commercial, and residential buildings owners. Several researchers proposed optimizing reservation and allocation systems [16,17,18,19]. 

### 2.2. Forecasting Using Recurrent Neural Network

The parking demand forecast is vital to shared parking planning and management. Jiang and Zhang [20] built a parking demand prediction model using regression analysis. It shows that the predicting result is closer to the actual parking demand, reducing the imbalance between supply and demand and expanding parking space utilization. Moreover, some researchers utilize machine learning to develop forecasting models. For example, Zhao et al. [21] compared some machine learning methods to predict parking occupancy. The machine learning methods used include linear regression, support vector regression, neural network, and autoregressive integrated moving average. Badii et al. [22] also used Bayesian regularized neural network (BRNN) to predict parking space availability. 

**Figure 1:**
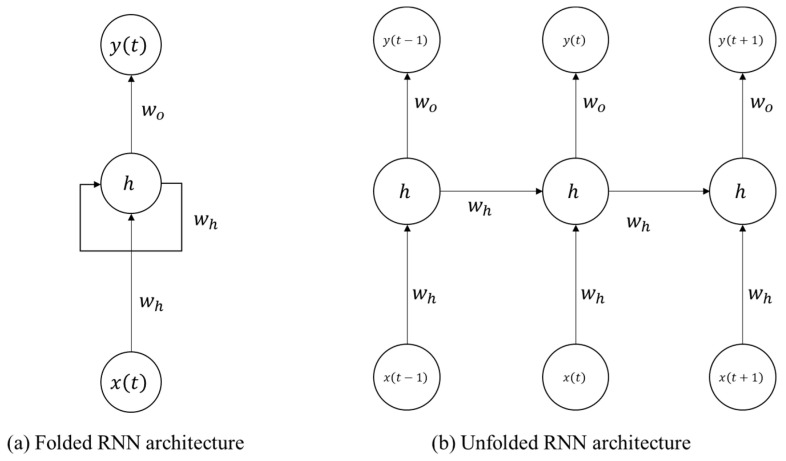
RNN architecture.

One of the rising forecasting methods is the recurrent neural network (RNN). RNN is a machine learning technique that computes new states on the basis of information from previous sequence steps. To predict output y(t), the RNN is trained based on input x(t) from a time series or temporal shift [23]. Figure 1 shows the architecture of a general RNN. It comprises an input, an output, and hidden nodes connected as weighted, directed, and cyclic graphs. In contrast to other neural networks using different parameters in each layer, the RNN shares the same weights in every time step. Some studies have proposed learning procedures in the training process [24,25,26].

Many applications have implemented forecasting using the RNN. For example, Kong et al. [27] applied the RNN to forecast energy consumption in residential areas. This study employed an RNN framework to manage high volatility and uncertainty in the data. Likewise, Chang et al. [28] forecasted water levels to mitigate future flood damage. In the transportation management field, Chen et al. [29] used RNN architecture to predict station level demand in a bike sharing system in the transportation field. In the proposed system, the RNN is applied to predict the number of internal usages of the parking spaces in open-to-specific-group parking lots.

### 2.3. Internet of Thing and Cloud Computing

Internet of Things (IoT) connects a wide range of industrial and consumer devices, thus generating a large volume of data for analysis, and is essential for cloud computing [29,30,31,32]. Furthermore, the interconnection of all these devices to produce more granular datasets improves data access and analysis [33].

Cloud storage and management obviates the need to purchase powerful computers to store and process the data they are interested in analyzing. Instead, users just need to rent a service from specialized companies or businesses to manage the datasets and provide real-time analysis based on user specifications [34,35]. Such data can be accessed through commonly used devices through the installation of specialized software or applications. Moreover, low-cost cloud services, such as computing and storage, motivate end-users of small/medium/large businesses and government agencies to use such platforms [36].

Recent studies have examined the exploitation capabilities of fog computing at the edge of the Internet to assist IoT in reducing mandatory connectivity requirements and to support computation or storage closer to where data are generated in real-time and its requirements [37,38,39]. Fog computing aims to employ numerous highly distributed edge nodes—such as sensors, actuators, routers, and microdata centers—to assist time-dependent, geographically distributed, or device/mobile applications [37,40]. Fog constructs are a powerful enabling complement to the IoT and cloud scenario and represent a new layer of collaborating devices that can execute services and complete exact business assignments [41].

## 3. Methodology

**Figure 2:**
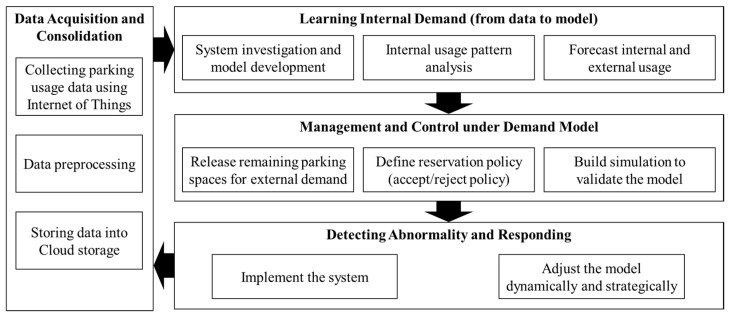
Framework of the proposed shared parking system.

The proposed shared parking system can solve the parking problem in cities by optimizing the utilization of available parking lots. This study focused on open-to-specific-group parking lots, such as parking lots at schools and offices. The proposed shared parking system comprises four primary steps: The first step includes data collection and data preprocessing. The proposed system applies an IoT system to collect internal usage data. In the second step, internal usage data are then analyzed to obtain an internal usage pattern, the future internal demand is predicted using the LSTM RNN, and the parking lots predicted to be unused are released to the public. The third step involves reservations, wherein parking lots released to the public can be used by anyone through the reservation system. People with a reserved parking space can then enter the parking lot. The final step focuses on continuous improvement. The data used to develop the prediction model are continuously updated in real-time to obtain accurate internal usage prediction. Figure 2 presents the framework of the proposed shared parking system.

### 3.1. System Investigation and Model Development Using Recurrent Neural Network

**Figure 3:**
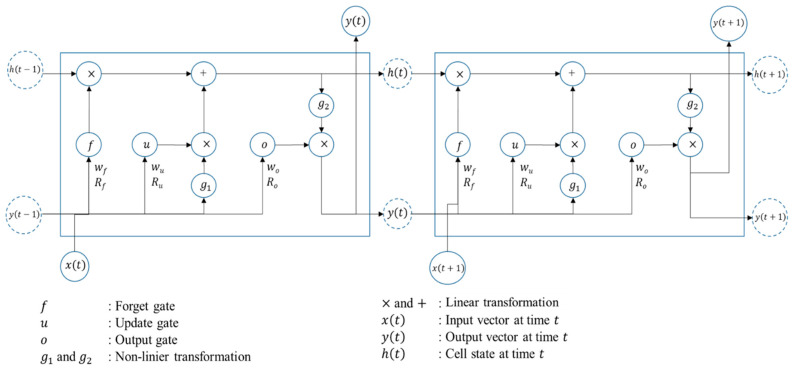
LSTM RNN Structure.

To fulfill internal and external demand, the proposed system must be able to predict internal usage before releasing the spaces for public use. This study predicted internal usage using the LSTM RNN (Figure 3). The LSTM RNN architecture assists the system in storing the data pattern derived over a long period by using efficient approaches. The system has open and close gates, which decide the information to be stored in, written to, and read from a node. The LSTM RNN architecture in Figure 3 has a forget gate, update gate, and output gate.

Computations of each gate at time t follow Equations (1)–(3) when the cell state is updated based on Equation (4). Where $W_{f},\;W_{u},\;W_{h}$, and $W_{o}$ are weights from an input node to the forget gate, update gate, candidate cell state, and output gate, respectively. $R_{f},\;R_{u},\;R_{o}$, and $R_{h}$ are recurrent weights from the input node to forget gate, update gate, output gate, and cell state, respectively. $b_{f},\;b_{u},\;b_{o}$, and $b_{h}$ are bias vectors. Function σ can be set as sigmoid, hyperbolic tan, and other functions.

(1) $$f\left(t\right)=\sigma \left(W_{f}x\left(t\right)+R_{f}y\left(t-1\right)+b_{f}\right)$$

(2) $$u\left(t\right)=\sigma \left(W_{u}x\left(t\right)+R_{u}y\left(t-1\right)+b_{u}\right)$$

(3) $$o\left(t\right)=\sigma \left(W_{o}x\left(t\right)+R_{o}y\left(t-1\right)+b_{o}\right)$$

(4) $$h\left(t\right)=u\left(t\right)⊙\tilde{h}\left(t\right)+\sigma_{f}\left(t\right)⊙h\left(t-1\right)$$

where $\tilde{h}\left(t\right)$ is a candidate state given by using Equation (5)

(5) $$\tilde{h}\left(t\right)=g_{1}\left(W_{h}x\left(t\right)+R_{h}y\left(t-1\right)+b_{h}\right)$$

Finally, the output is given by Equation (6)

(6) $$y\left(t\right)=\sigma_{o}\left(t\right)⊙g_{2}\left(h\left(t\right)\right)$$

**Figure 4:**
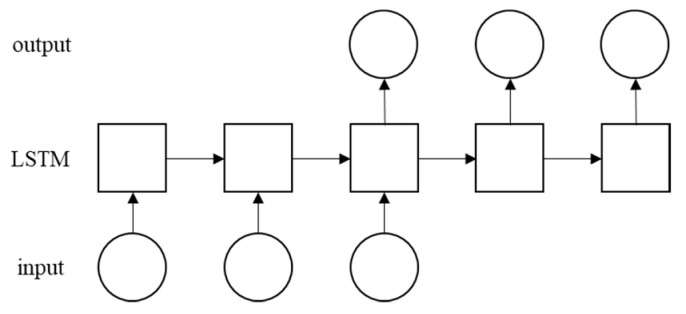
Many-to-many LSTM RNN Architecture.

Figure 4 shows a many-to-many architecture used as the LSTM RNN architecture in this study. This figure shows that n inputs are provided to a hidden layer comprising the number of nodes. It then provides m outputs. The output is m period predicted by the system. In the proposed system, n input represents the data size in one session. 

### 3.2. Management and Control Based on Model

**Figure 5:**
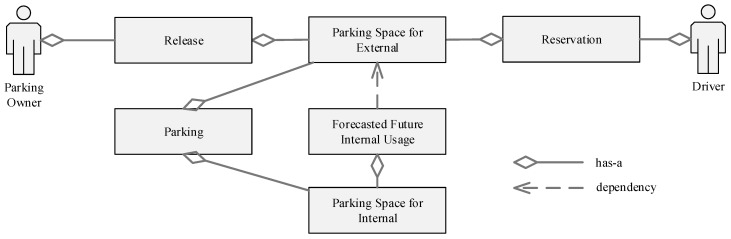
Domain modeling diagram describing the requirement needs.

The proposed system requires the real-time and continuous information on parking usage, number of predicted internal usage, and number of parking reservations. Figure 5 shows requirements that must be captured. It shows that the predicted future internal usage is necessary to decide the number of parking released. Parking owners can be the owner of the parking space or the parking management, and the driver is a public user who wants to reserve a parking space (external demand). 

**Figure 6:**
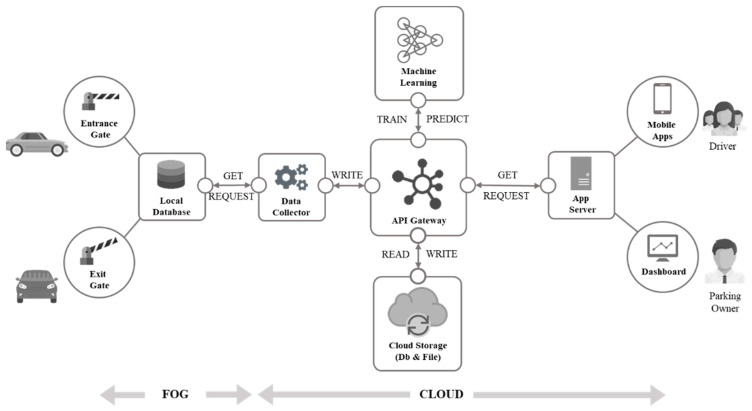
Cloud-based architecture system.

To implement the aforementioned requirements, an architecture system was designed based on fog and cloud computing (Figure 6). To obtain daily information parking usage, in each parking area, fog computing was initially implemented by collecting data from a parking sensor. The system collected the car data that entered and exited the parking lot when the driver swiped their card to open the entrance and exit gates. The raw data was then preprocessed to obtain hourly usage and uploaded to the local database.

A server-side data-processing pipeline simultaneously processed data from the local parking database, which is saved in a cloud storage system. To determine how many parking spaces can be released, the system must predict the internal usage because the space can be reserved for at most the subsequent 7 days. The system, which applies machine-learning techniques, was executed every day at 23:00. The prediction result is stored in the cloud storage. Finally, this parking system uses an application programming interface gateway between the server and the client.

**Figure 7:**
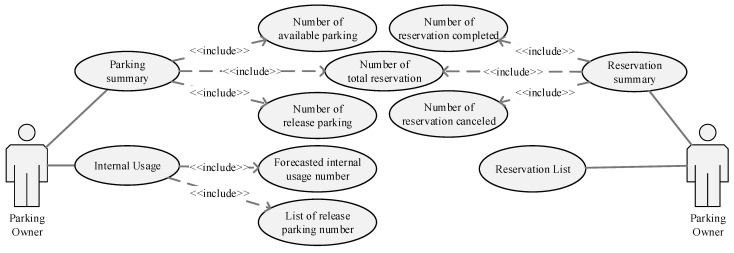
Use case diagram of parking dashboard for parking owner.

**Figure 8:**
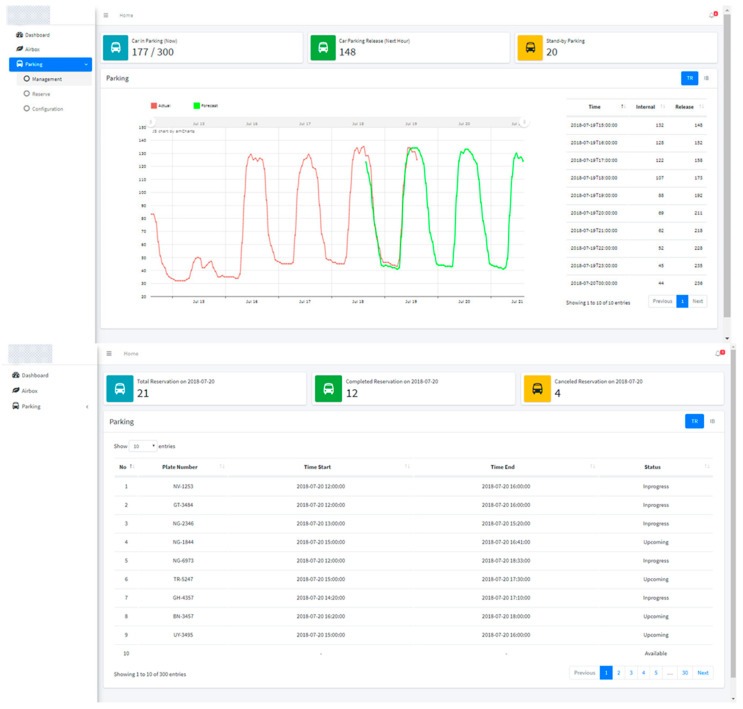
Implementation of parking dashboard using web platform.

The proposed system offers a dashboard application and a reservation application to mediate between the parking owner and the driver. The parking dashboard is a web-based app that assists the parking owner to track reservations and develop a booking schedule. The dashboard presents real-time parking information such as the number of parking spaces available, the number of parking spaces released, predicted internal usage, and parking released for the next few hours. Moreover, the dashboard assists the owner to monitor reservations by displaying the number and status of reservations. Figure 7 and Figure 8 present the case diagram and interface of the parking dashboard, respectively.

**Figure 9:**
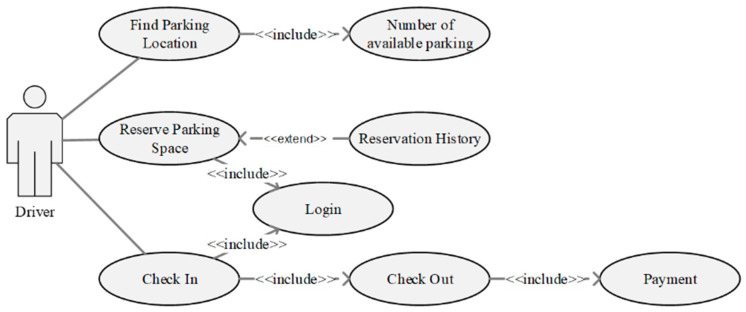
Reservation use case diagram for driver.

**Figure 10:**
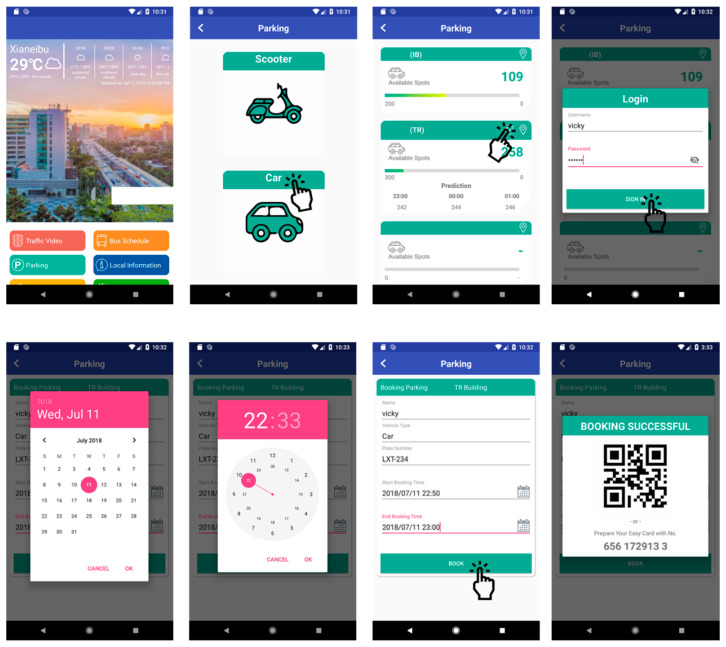
Implementation of reservation based on mobile platform.

The reservation application is a mobile-based application developed to assist drivers with obtaining information regarding parking space availability and reservations (Figure 9). The system generates a quick response (QR) code as an e-ticket after the successful reservation of a parking space. If the driver uses a contactless payment card, they must register the identity (ID) card during registration. The system allows the car owner or driver to check in and open the entrance gate by using the QR code in the e-ticket or contactless payment card at least 5 min before the reserved time. Figure 10 shows the implementation of the mobile reservation application.

### 3.3. Abnormality Detection and Response

**Figure 11:**
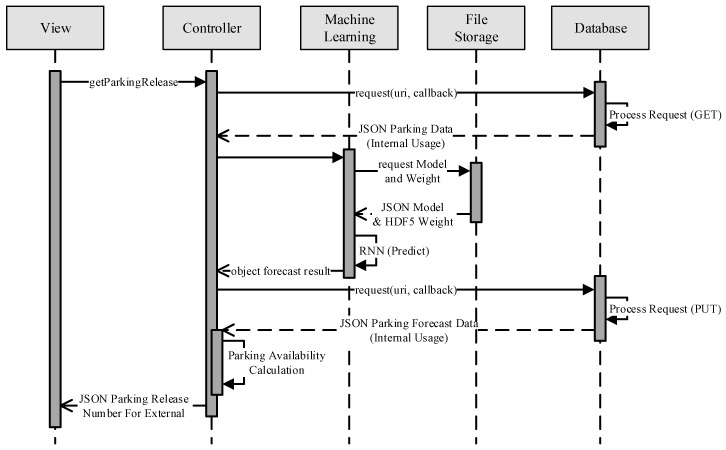
Sequential diagram of forecasting process.

The proposed system continuously collects real-time data and makes decisions based on the latest data. To maintain the accuracy of prediction and to smoothly run the system, prediction for the next 7 days is always updated every day (Figure 11). Reservations can thus only be made a maximum of 7 days in advance. This updating schedule helps quickly respond to any abnormalities. The proposed system utilizes the prediction to recommend the parking space owner to release their parking space. However, the parking space owner still manually set the releasing process. Figure 11 describes the sequence diagram of this process. This module was developed using Keras, which is a high-level neural networks API written in Python which can run on TensorFlow [42,43]. It helps to implement RNN. 

## 4. Simulation and Application 

As a case study, this study applied the proposed system to a parking lot owned by a university in Taipei City. The university has some parking lots intended for use by teachers, staff, and students. Elementary and junior high schools, markets, and some offices surround the university. During lunch or dinnertime, finding parking lots around the university becomes difficult. This case study simulates the benefit of using the proposed shared parking system in the university. 

### 4.1. Data Collection

Internal usage data is collected through a system installed at the gate of the parking lot. The system records data when a driver swipes their ID card over a machine at the gate. Approved drivers can enter the parking area. Table 1, Table 2 and Table 3 present the attributes of the collected data. To determine the use pattern, hourly data is processed to display the number of parking used.

**Figure 12:**
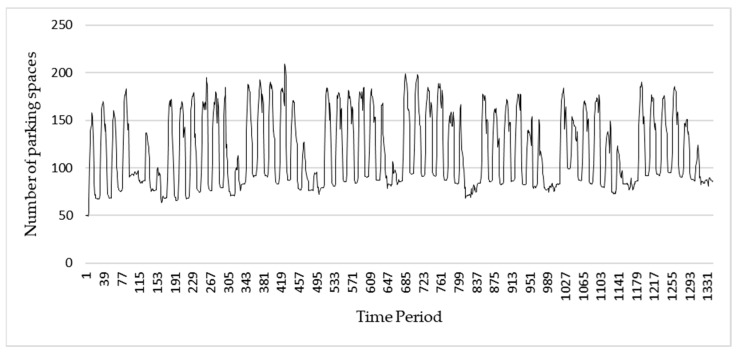
Parking data.

Real-time parking data was collected from 1 July 2016 to 31 October 2016. Figure 12 shows that in general, parking use has a weekly cycle. A daily cycle was also seen, particularly during weekdays. Per this pattern, the LSTM RNN architecture used in this system has 168 inputs (hourly data for 7 days) as well as output prediction for the subsequent 168 periods. Therefore, the system can predict usage for the subsequent 7 days. 

### 4.2. Prediction Results

**Figure 13:**
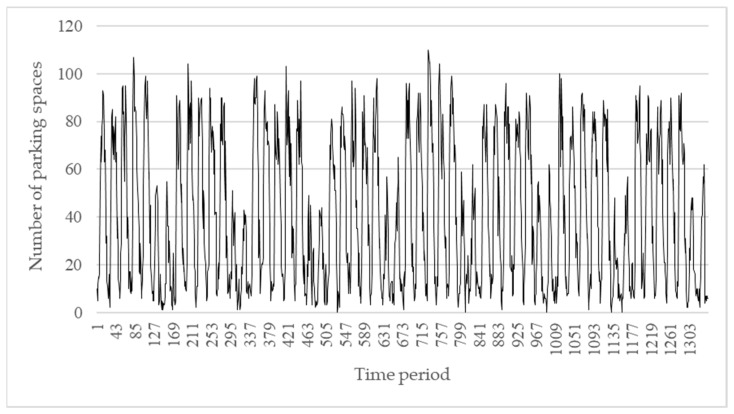
Artificial dataset.

After data collection, the proposed system uses the available data to make predictions. To evaluate the proposed prediction algorithm, some simulations were performed using the real-time data and artificial datasets. This study uses an artificial dataset as an additional dataset because the real data in only available for four months of data. The artificial dataset was generated on the basis of Poisson distribution using a dynamic lambda value. The lambda value was periodically changed to follow the pattern of the real-time data. Figure 13 shows the pattern of the artificial datasets.

The LSTM RNN algorithm involves some parameters. The parameters used in this study are presented in Table 4. The Look_back is a time window representing the number of previous data points used to predict the next value. The Look_back was set as 168 points at week 1 of data collection and was determined on the basis of historical data patterns of weekly cycles. A target is the number of predicted periods. This study predicted 168 h ahead of any given moment. The recurrent_activation argument was applied to the input, forget, and output gates. The value for this argument was determined using a sigmoid function. activation argument was applied to the candidate and output hidden states. The value of this argument was obtained using a sigmoid function. Optimizer is an algorithm used to optimize the LSTM RNN weights. This study applied a Nadam optimizer. Loss_function and epoch are accuracy measurements and the number of iterations for each model, respectively.

Parameter settings were determined on the basis of preliminary simulations conducted to obtain the improved parameter settings. To obtain the optimal prediction result, this study compared several forecasting algorithms, including seasonal ARIMA, support vector regression (SVR), multilayer perceptron (MLP), convolutional neural network (CNN), long short-term memory with ensemble empirical mode decomposition (LSTM-EEMD), LSTM RNN with a many-to-one structure, and LSTM RNN with a many-to-many structure. Table 5, Table 6, Table 7 and Table 8 summarize simulation results of these algorithms.

**Figure 14:**
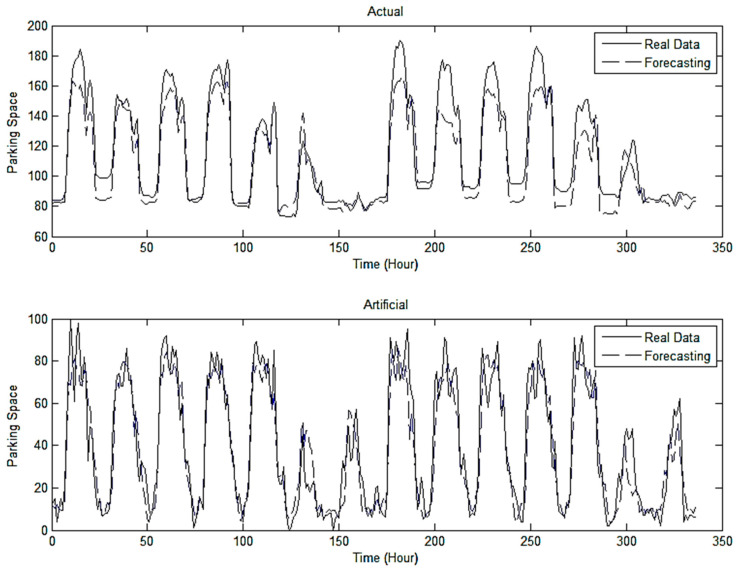
Pattern of forecasted real and artificial datasets.

These results show that the SVR algorithm obtained the smallest mean absolute and root mean square errors for a training set. However, the error rate for a testing error was significantly different for artificial and real-time datasets. The SVR algorithm is susceptible to overfitting. Therefore, although SVR gave the smallest training error, this study did not use SVR results. By contrast, among other algorithms with no overfitting, the LSTM RNN with the many-to-many structure was able to obtain the smallest testing error for artificial and actual datasets. Therefore, this study used forecasting results from the LSTM RNN with the many-to-many structure. Figure 14 shows prediction results for the artificial and real-time datasets, thus indicating that the error rates of prediction results obtained using the LSTM RNN with the many-to-many structure algorithm were relatively low. 

Table 9 shows the computational time of each algorithm. Because the LSTM RNN has a more complex procedure, it requires a longer time than seasonal ARIMA, SVR, and MLP algorithms. The LSTM RNN with the many-to-many architecture is faster than that with the many-to-one architecture.

### 4.3. System Simulation

**Figure 15:**
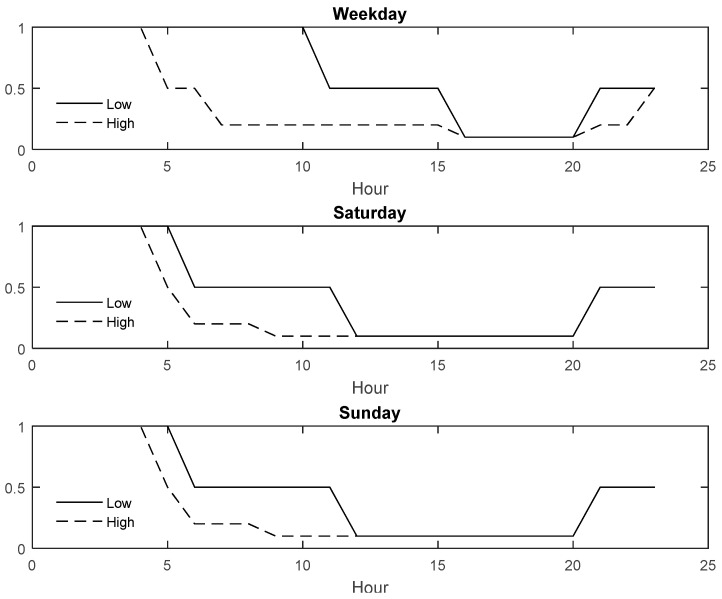
Lambda value for external demand scenario.

After predicting the internal usage, the system releases the remaining parking spaces for public usage. To evaluate the proposed system, two scenarios with low and high external demand were generated to make datasets. The external demand was generated on the basis of Poisson distribution with dynamic lambda. Dynamic lambda was used because, in real-time applications, the demand for the parking spaces varied after some time. Therefore, to mimic the real-time problem, this study generated the external demand data with dynamic lambda. Figure 15 illustrates the change in the lambda value over a period of time for low- and high-demand scenarios.

The next step involves simulating the acceptance process. The proposed system accepts reservations from the external demand if a parking space is available according to predictions. To evaluate the proposed shared parking system, this study compared the results with a static releasing scenario. In a static releasing scenario, the number of parking spaces released is always fixed. In this study, three fixed release scenarios were used, namely 20, 50, and 100 spaces. These numbers were determined on the basis of the real-time data of internal usage, which was 50–209 with a maximum capacity of 300 spaces. Without prediction, releasing 20–50 parking spaces did not disturb internal usage. To make a risky decision, decision makers could release up to 100 spaces. Therefore, these numbers were compared with the number of parking spaces released based on the prediction result.

Table 10 summarizes simulation results for all scenarios evaluated in this study. The revenue in this table is calculated using Equation (7).

(7) Revenue=H×R

where H is total hour of rented and R is the parking rate per hour. In this study, the parking rate is assumed as NT$30/h. This result indicates that for the artificial data, the proposed shared parking system could accept all reservations. Simulation results obtained using the real-time internal demand data show that the proposed system could generate more revenue when the external demand was high. This result was observed because the proposed system allowed the acceptance of more reservations to meet external demand without disturbing the internal demand. Moreover, 100 spaces were released to meet external demand, and thus the system accepted a high number of external demands. However, releasing 100 spaces to meet the external demand jeopardized the ability to meet the internal demand. The simulation results indicated that the proposed shared parking system is a favorable solution for the parking problem, particularly in cities.

## 5. Conclusions

The lack of parking spaces is a common problem in cities. In this study, a shared parking system was proposed as a solution for the urban parking problem. The proposed system solves the parking problem by optimizing the utilization of existing parking lots to increase the availability of parking spaces. The primary idea of the proposed shared parking system is to release several open-to-specific-group parking lots for public use without overriding personal use. Open-to-specific-group parking lots contain parking spaces provided for particular people, such as parking spaces in universities provided for teachers, staff, and students. Another example is parking spaces in an office building, which are generally used by people working in that building. The proposed shared parking system aims to increase the utilization of such parking lots by releasing unoccupied parking spaces to the public. The most challenging limitation of releasing several open-to-specific-group parking lots for public use is ensuring that the requirements of the group are not disturbed.

The proposed shared parking system comprises four primary steps, namely data collection and data preprocessing using an IoT system, predicting internal demand by using a LSTM RNN algorithm, releasing unoccupied parking lots based on prediction, and continuously updating the real-time data and predictions. This study used a parking space owned by a university in Taiwan as a case study. The proposed system was evaluated using the artificial and real-time datasets. To evaluate the effectiveness of the proposed system, the proposed shared parking system was examined under different scenarios in which a fixed number of parking spaces were released. Simulation results indicated that the proposed shared parking system can accommodate greater public usage and allow parking management to yield additional profit from releasing parking spaces to the public without compromising internal usage. This result indicates that the proposed shared parking system is a favorable solution to the parking problem in cities.

## Figures and Tables

**Table 1:** The attribute of data collected.

Attribute Name	Data Type	Description
Member ID	VARCHAR	Driver identity number, e.g., “Z532”, “A81214”.
Department name	VARCHAR	Driver department name, e.g., “school of management”, “administrative unit”.
Check-in/check-out time	DATETIME	Date and time when the car entrance or exit gate’s machine, e.g., “31 December 2016 23:51:26”.
Building name	VARCHAR	Parking location name.
Machine card	VARCHAR	Entrance or exit machine to open the gate. “A” for entrance gate, “B” for exit gate.
Update time	DATETIME	Log date and time when data is inserted to database.

**Table 2:** Example data of parking used.

Date Time	Weekend	Day	Parking Used
8 July 2016 19:00	0	6	87
8 July 2016 20:00	0	6	85
8 July 2016 21:00	0	6	84
8 July 2016 22:00	0	6	85

**Table 3:** Attributed description of data after data preprocessing.

Attribute Name	Data Type	Description
Date and time	DATETIME	Date and time in hourly
Weekend	INT	Weekend flag, “1” for weekend or “0” for weekday.
Day	INT	Day in number, “1” for Sunday, “7” for Saturday, “2–5” for Monday to Friday.
Parking used	INT	Number of parking used by internal usage.

**Table 4:** Parameter setting for RNN with many-to-many structure.

No.	Parameter	Value
1.	Look back	168
2.	Target	168
3.	Activation	sigmoid
4.	Recurrent activation	sigmoid
5.	Optimizer	Nadam
6.	Loss_function	mse
7.	Epoch	1500

**Table 5:** Mean absolute error for artificial dataset.

Method	Set	Average Value	Minimum Value	Maximum Value	Standard Deviation
Seasonal ARIMA	Training	14.148	NA		
	Test	13.644			
Support vector regression	Training	**0.132** *	NA		
	Test	8.541			
Multi-layer perceptron	Training	1.552	0.399	5.461	1.570
	Test	8.283	7.595	10.173	0.724
Convolutional neural network	Training	0.580	0.221	0.867	0.238
	Test	10.012	9.711	10.319	0.190
LSTM-EEMD	Training	17.750	16.628	19.368	1.435
	Test	18.308	17.658	19.382	0.937
LSTM RNN many-to-one	Training	15.591	8.107	29.257	6.113
	Test	17.447	8.327	29.060	7.196
LSTM RNN many-to-many	Training	8.521	8.519	8.524	0.001
	Test	**7.984** *	7.965	7.990	0.007

*: Best result.

**Table 6:** Mean absolute error for actual dataset.

Method	Set	Average Value	Minimum Value	Maximum Value	Standard Deviation
Seasonal ARIMA	Training	20.997	NA		
	Test	18.576			
Support vector regression	Training	**0.145** *	NA		
	Test	15.963			
Multi-layer perceptron	Training	2.856	0.974	5.107	1.387
	Test	23.288	18.975	25.513	2.165
Convolutional neural network	Training	1.884	0.784	4.002	0.981
	Test	13.562	12.368	16.159	1.074
LSTM-EEMD	Training	33.361	31.016	35.075	2.102
	Test	25.549	24.533	27.316	1.536
LSTM RNN many-to-one	Training	25.489	19.370	30.956	3.836
	Test	24.728	17.486	29.265	3.859
LSTM RNN many-to-many	Training	11.123	11.117	11.144	0.008
	Test	**7.990** *	7.931	8.130	0.056

*: Best result.

**Table 7:** Root mean square error for artificial dataset.

Method	Set	Average Value	Minimum Value	Maximum Value	Standard Deviation
Seasonal ARIMA	Training	18.358	NA		
	Test	17.974			
Support vector regression	Training	**0.159** *	NA		
	Test	11.035			
Multi-layer perceptron	Training	1.859	0.514	6.473	1.822
	Test	10.779	9.910	13.207	0.921
Convolutional neural network	Training	0.770	0.296	1.169	0.321
	Test	12.947	12.504	13.290	0.234
LSTM-EEMD	Training	21.642	20.282	23.424	1.612
	Test	22.064	21.309	23.378	1.141
LSTM RNN many-to-one	Training	21.829	10.247	38.096	7.806
	Test	23.635	11.165	37.080	8.469
LSTM RNN many-to-many	Training	11.044	11.000	11.050	0.015
	Test	**10.405** *	10.397	10.408	0.003

*: Best result.

**Table 8:** Root mean square error for actual dataset.

Method	Set	Average Value	Minimum Value	Maximum Value	Standard Deviation
Seasonal ARIMA	Training	27.869	NA		
	Test	25.234			
Support vector regression	Training	**0.189** *	NA		
	Test	20.974			
Multi-layer perceptron	Training	3.862	1.319	7.198	1.753
	Test	30.337	24.150	33.331	2.883
Convolutional neural network	Training	2.590	1.065	5.215	1.261
	Test	18.794	16.779	23.741	1.998
LSTM-EEMD	Training	40.744	38.397	41.926	2.033
	Test	30.565	29.613	32.303	1.507
LSTM RNN many-to-one	Training	35.872	29.172	42.340	4.107
	Test	33.418	24.669	40.283	4.528
LSTM RNN many-to-many	Training	15.372	15.363	15.385	0.008
	Test	**10.597** *	10.511	10.795	0.079

*: Best result.

**Table 9:** Computational time(s).

Seasonal ARIMA	Support Vector Regression	Multi-Layer Perceptron	Convolutional Neural Network	Lstm-Eemd	Rnn Lstm Many-To-One	Rnn Lstm Many-To-Many
13.44	3.81	124.77	171.01	1650.83	8341.55	255.71

**Table 10:** Simulation result.

Release Type	Internal Demand	Lambda External Demand	Number of Reservations Accepted	Acceptance Ratio	Revenue (in NT$) *
Dynamic: based on forecasting result	Artificial data	High	1786	100%	339,990
Dynamic: based on forecasting result	Artificial data	Low	1125	100%	219,510
Dynamic: based on forecasting result	Real data	High	1758	98%	334,410
Fix: 20 spaces released	Real data	High	786	44%	138,990
Fix: 50 spaces released	Real data	High	1367	77%	250,080
Fix: 100 spaces released	Real data	High	1650	92%	310,410
Dynamic: based on forecasting result	Real data	Low	1125	100%	219,510
Fix: 20 spaces released	Real data	Low	645	57%	116,520
Fix: 50 spaces released	Real data	Low	1036	92%	200,250
Fix: 100 spaces released	Real data	Low	1125	100%	219,510

*: With assumed parking rate NT$30/h. Revenue = Total hour of rented × parking rate/hour.

## Data Availability

Not applicable.
